# Generation and characterization of an *Advillin*-Cre driver mouse line

**DOI:** 10.1186/1744-8069-7-66

**Published:** 2011-09-11

**Authors:** Sandra Zurborg, Agnieszka Piszczek, Conception Martínez, Philip Hublitz, Mumna Al Banchaabouchi, Pedro Moreira, Emerald Perlas, Paul A Heppenstall

**Affiliations:** 1 Mouse Biology Unit. European Molecular Biology Laboratory (EMBL), Via Ramarini 32, 00016 Monterotondo (Roma), Italy

## Abstract

Progress in the somatosensory field has been restricted by the limited number of genetic tools available to study gene function in peripheral sensory neurons. Here we generated a Cre-driver mouse line that expresses Cre-recombinase from the locus of the sensory neuron specific gene *Advillin*. These mice displayed almost exclusive Cre-mediated recombination in all peripheral sensory neurons. As such, the Advillin-Cre-driver line will be a powerful tool for targeting peripheral neurons in future investigations.

## Background

The identification of new molecular targets and mechanisms in the peripheral nervous system is an on-going challenge in the field of somatosensation. Our understanding of this system has often been limited by the ubiquitous expression of many sensory neuron genes and an inability to target these genes specifically in peripheral sensory neurons.

Knockout mice are the tools of choice for studying mammalian gene function, but perinatal lethality and developmental compensation are commonly occurring problems that can complicate interpretation of results. These can be avoided by creating tissue- or developmentally specific knockout mice by means of the Cre-*loxP *system, where specific Cre-recombinase driver mice are crossed with mice containing *loxP*-flanked ('floxed') genes of interest [1,2].

Several Cre mouse lines are available that target subpopulations of neurons in the peripheral sensory system, in particular nociceptive neurons. These include mice where Cre is under control of the locus of the voltage-gated sodium channel Na_v_1.8 (expression in most nociceptors but not in mechanoreceptive neurons) [3-6], or of the *Peripherin *gene, a marker for small sensory neurons [7]. Similarly, many Cre-driver mouse lines are available for targeting neuronal subpopulations in the central nervous system. However, to date it has not been possible to generate Cre drivers to target all subpopulations of peripheral sensory neurons without affecting other tissues in the organism. Hence, the existence of Cre mouse lines with specificity for the peripheral nervous system (PNS) would allow a much more detailed investigation of the molecular mechanisms and function of primary sensory neurons.

Advillin is an actin regulatory/binding protein of the gelsolin/villin family with a role in neuronal outgrowth and stress response in peripheral sensory neurons [8-11]. The initial gene-expression study of *Advillin *by Northern blot analyses and *in situ *hybridization revealed that it is found in uterus, testis, taste buds, and also at low levels in the brain [12]. Strong expression was found in dorsal root ganglia (DRG) and trigeminal ganglia (TG) at embryonic stages E14.5 to E16.5 and in the adult animal. Hasegawa and colleagues [9] created an alkaline phosphatase knock-in mouse line of the Advillin gene locus (*Avil-hPLAP *mice) in order to perform histological analyses. They found *Advillin *expression almost exclusively in peripheral sensory neurons during development and adulthood and observed strong staining in DRG, TG, vestibulocochlear ganglia, plossophayrngeal ganglia, and vagus ganglia. In line with this finding, the expression of *Pervin*, which is the rat homologue of the mouse Advillin protein, is restricted to DRG neurons and superior cervical ganglia neurons [8,10,13]. Hence, Advillin appears to be a promising candidate for the generation of a Cre-driver mouse line with specificity in the PNS ganglia.

Here we report the generation and characterization of BAC-transgenic animals expressing Cre-recombinase under the regulatory elements of the *Advillin *gene. We present the pattern of specific Cre-mediated recombination by means of β-galactosidase staining after crossing with *lacZ *reporter mice containing a LoxP-flanked STOP-cassette upstream of a LacZ expression cassette [14]. Furthermore, we investigate acute pain behavior in these mice. Our data indicate that this mouse line is a valuable tool to specifically target and investigate sensory neurons in dorsal root ganglia and trigeminal ganglia.

## Results

### Generation of Advillin-Cre transgenic mice and copy number analysis

**Figure 1:**
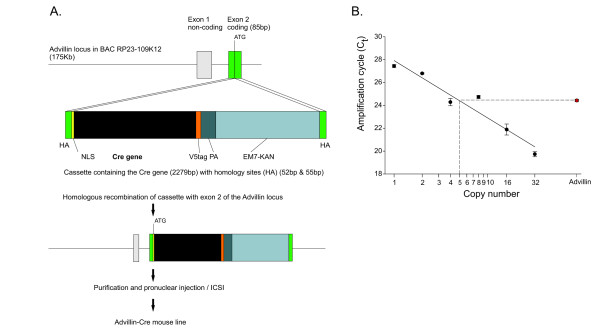
**Generation of Advillin-Cre transgenic mice**. **A**. Schematic representation of ET-recombination based insertion of a NLS-Cre gene cassette into the coding region of a BAC containing the mouse *Advillin *gene locus. The cassette was recombined into the locus immediately after the start codon of exon 2. After purification, the final 177 kb construct was injected into the pronucleus or used for intracytoplasmic sperm injection. HA: homology arms for recombination; NLS: SV-40-T nuclear localization signal; Cre: Cre gene; V5: V5 epitope tag; PA: SV-40 polyadenylation signal; EM7-KAN: kanamycin cassette under a prokaryotic EM7 promoter. **B**. Copy number analysis by means of *LightCycler *PCR revealed that Advillin-Cre mice have five copies per genome.

We chose a bacterial artificial chromosome (BAC) of the RP23 library (RP23-109K12) that contains a total of 175 kb, and thereof 168 kb of upstream putative regulatory sequence to the *Advillin *gene. The BAC clone was verified and modified by lambda RedET-recombination (homologous recombination in *Escherichia coli*) to incorporate a cassette containing the SV40 T nuclear localization signal, the *Cre-*gene, and the SV40 large T antigen polyA signal (Figure 1A) [15,16]. The homology arms necessary for homologous recombination were introduced to the cassette as described in *Materials and Methods*, and the NLS-Cre-pA cassette was inserted into exon 2 of the *Advillin *locus, thereby substituting the endogenous ATG of *Advillin *with the NLS-Cre coding sequence (Figure 1A). The outcome of recombineering was screened by restriction digest and PCR, and after obtaining successfully modified clonal colonies, integrity of the introduced cassette was verified by full length sequencing. Afterwards, the DNA of the Advillin-Cre modified BAC was isolated, purified, and either microinjected into the pronucleus of oocytes from B6D2F1/N mice (Charles River Laboratories) or prepared for intracytoplasmic sperm injection (ICSI), where the DNA is injected together with membrane-damaged sperm cells into non-fertilized metaphase II oocytes [17,18]. Both applications successfully produced transgenic Advillin-Cre animals.

There is a strong correlation between increased copy number integration and increased transgene expression in independent transgenic lines [19,20]. BAC transgenic mice generally do not contain more than 1-5 copies, although rarely, some transgenic animals with more copies have been achieved [19,21-23]. To determine the situation in our generated animals, we compared a sample of unknown copy number from our Advillin-Cre mouse genomic DNA to a range of DNA copy number standards. We spiked in varying amounts of purified BAC DNA into wildtype mouse genomic DNA and by means of *LightCycler *qPCR amplification cycle values were plotted to generate a standard curve to estimate the copy number from our Advillin-Cre mice. Thereby, we calculated that our described Advillin-Cre line has five copies of the transgene per genome (Figure 1B).

### Cre is expressed in peripheral sensory tissues in adult Advillin-Cre mice

We mated heterozygous male Advillin-Cre^tg/+ ^mice with homozygous female Rosa26-*lacZ*^tg/tg ^reporter (R26R) mice [14] (Jackson Laboratories, Bar Harbor, ME) in order to characterize the expression pattern of functional Cre. The reporter mice carry a stop cassette flanked by *loxP *sites preceding the gene for β-galactosidase. The latter will be selectively expressed in the presence of active Cre recombinase that excises the floxed polyadenylation site. In addition, we crossed male Advillin-Cre^tg/+ ^mice with a novel, as yet unpublished, Rosa26-CFP reporter mouse strain.

**Figure 2:**
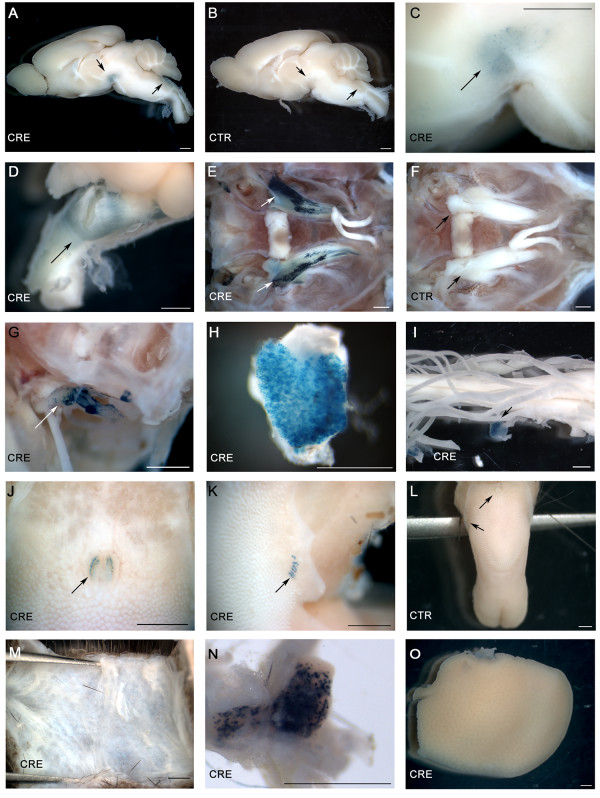
**X-gal staining in whole-mount tissues from adult mice**. Cre mediated recombination was detected in adult Advillin-Cre mice crossed with R26R mice for *lacZ *expression. Organs were collected and stained with X-gal. Representative pictures are shown. **A**. brain from Cre expressing mice and **B**. Cre negative animals. **C**. close up of midbrain region of **A**. **D**. brainstem. **E**. and **F**. trigeminal ganglia from Advillin-Cre mice and littermate controls, respectively. **G**. vestibular ganglion. **H**. DRG. **I**. unstained spinal cord with a stained DRG. **J**. circumvallate taste buds in the tongue. **K**. foliate taste buds in the tongue. **L**. tongue from control mice. X-gal negative **M**. skin **N**. superior cervical ganglia, and **O**. liver from Cre expressing mice. **CRE: **Advillin-Cre mice. **CTR: **wildtype littermates controls. Scale bar: 1 mm.

We collected adult tissues from the progeny in order to screen for Cre recombinase expression and Cre^+/+^-*lacZ*^tg/+ ^littermates were used as a negative control. The expression of functional Cre was investigated by staining for β-galactosidase (*lacZ *activity) using X-gal as described previously [14]. X-gal was detected in whole-mount tissues, such as the midbrain (Figure 2A and 2C), brainstem (Figure 2A and 2D), tongue (Figure 2J and 2K), vestibular ganglia (Figure 2G), trigeminal ganglia (Figure 2E), dorsal root ganglia (Figure 2H), superior cervical ganglia (SCG) (Figure 2N), and nodose ganglia (not shown). Figure 2B, F, and 2L depict brain, trigeminal ganglia, and tongue, respectively, from control animals. We did not observe any Advillin expression in olfactory or visual ganglia, other CNS regions or non-neuronal tissues. Thus, spinal cord (Figure 2I), liver (Figure 2O), skin (Figure 2M), thymus, testes, heart, and ovaries (not shown) were Cre negative. Intestines were excluded from the study due to endogenous β-galactosidase expression in Cre negative reporter mice.

**Figure 3:**
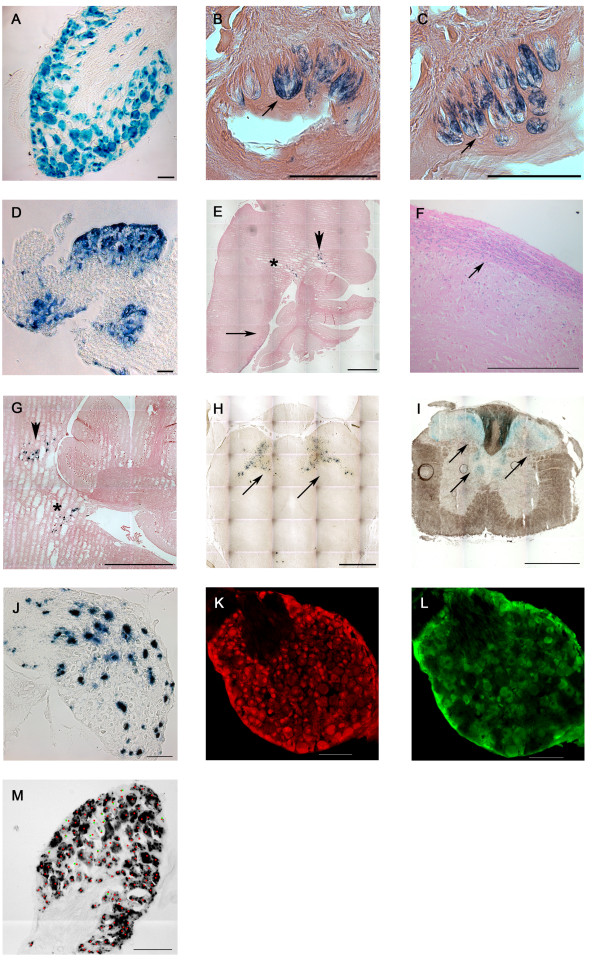
**X-gal staining in tissue sections from adult mice**. Cryo-sections from adult tissue after staining with X-gal, NeuN, or GFP. **A**. DRG. **B**. circumvallate taste buds in the tongue. **C**. foliate taste buds in the tongue. **D**. trigeminal ganglion. **E**. sagittal brainstem section. **F**. close up of caudal brainstem with staining in spinal tract of trigeminal nerve and spinal nucleus of trigeminal nerve (arrow). **G**. close up of rostral brainstem region with some staining in the medial trigeminal nucleus (arrowhead) and medial vestibular nucleus (star). **H**. Midbrain section with diffuse staining in the ventral tegmental area. **I**. coronal brainstem section with staining in secondary sensory ganglia. **J**. SCG section. **K**. and **L**. DRG sections with NeuN staining (red) and GFP staining (green) for quantification. **M**. DRG section with X-gal staining illustrating the quantification procedure. Scale bar: A. and D. 10 micrometer. B., C., F., G., J., K., L., and M. 100 micrometer. E., H., and I. 1 mm.

A more detailed analysis by sectioning tissues (20 μm), that showed X-gal staining previously, revealed that the pattern of *β-galactosidase *expression was similar in trigeminal and dorsal root ganglia in Advillin-Cre^tg/+^/*lacZ*^tg/tg ^mice (Figure 3D and 3A, respectively). Most importantly, almost all neurons in these ganglia were stained.

We quantified DRG sections by staining the tissue of Advillin-Cre^tg/+^/CFP reporter mice. After double staining with anti-NeuN and anti-GFP antibodies, six sections were counted. 82.28% (743/903) of NeuN-positive cells (Figure 3K), and thus neuronal cells, were also positive for CFP (Figure 3L), and thus Cre expressing. Moreover, 87.74% (673/767) of neurons from six DRG sections of Advillin-Cre^tg/+^/*lacZ*^tg/tg ^mice, counted by their characteristic shape, were also positively stained for X-gal (Figure 3M).

Midbrain sections showed the ventral tegmental area (VTA) to be β-galactosidase positive (Figure 3H). In the tongue, the circumvallate and foliate papillae, which are both innervated by the glossopharyngeal nerve, expressed functional Cre (Figure 3B and 3C), while the fungiform papillae were X-gal-negative. Sagittal (Figure 3E, F, and 3G) and coronal (Figure 3I) sections of the brainstem were also taken and X-gal staining demonstrated some Cre activity. Sensory brainstem nuclei expressed *Advillin *sparsely (Figure 3E and 3I), such as the spinal (Figure 3E arrow, Figure 3F arrow, and I) and medial vestibular nucleus (Figure 3E and 3G star, Figure 3I), the nucleus of solitary tract (Figure 3I), the spinal nucleus of the trigeminal nerve (Figure 3I), and the medial trigeminal nucleus (Figure 3E and 3G arrowhead, Figure 3I).

In conclusion, *Advillin *is expressed mainly in peripheral sensory ganglia, with additional expression in selected cells of the taste buds, vestibular ganglia and second-order nuclei, as well as the ventral tegmental area of the midbrain.

### Cre expression during development

**Figure 4:**
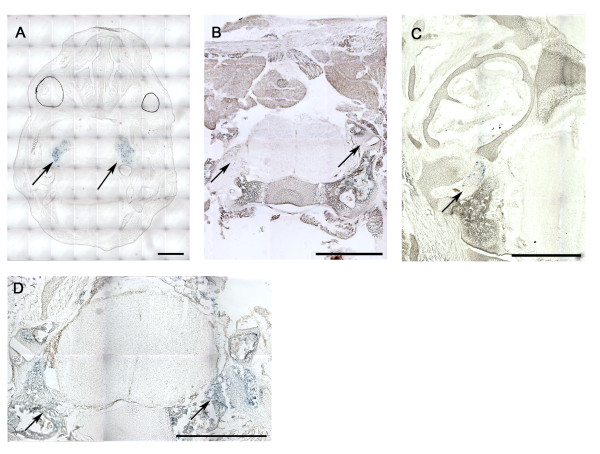
**X-gal staining for *lacZ *activity at embryonic stages and P1**. Sections from embryos expressing Advillin-Cre and *lacZ *during different stages. **A**. trigeminal ganglia at E16.5. **B**. unstained DRG and traces of endogenous β-galactosidase at E18.5. **C**. vestibular ganglion at E18.5. **D**. perinatal (P1) stained DRG section. Scale bar: 1 mm.

Timed matings were set up between heterozygous male Advillin-*Cre*^tg/+ ^mice and homozygous female Rosa26-*lacZ*^tg/tg ^reporter (R26R) mice [14] (Jackson Laboratories, Bar Harbor, ME). Females were sacrificed in accordance of animal welfare procedures to collect embryos at stages E12.5, E16.5, and E18.5. *Cre*^+/+^/*lacZ*^tg/+ ^embryo littermates were used as negative control. *Cre *expression was not detectable in any tissues at E12.5 (not shown), but trigeminal ganglia showed lacZ activity at E16.5 (Figure 4A) and E18.5 (not shown). We detected some endogenous β-galactosidase staining in the bone marrow of Advillin-Cre expressing animals (Figure 4C, E18.5), which was also observed in embryos from the control background. DRGs did not express *Advillin *at any embryonic stage (see Figure 4B for X-gal staining at stage E18.5). Therefore, we cut DRG sections from perinatal *Cre*^tg/+^/*lacZ*^tg/+ ^mice (P1) and demonstrated positive X-gal staining (Figure 4D). The vestibular ganglion expressed β-galactosidase at embryonic stage E18.5 (Figure 4C), while the remaining central nervous system and tissues were Advillin-Cre negative during development.

### *Advillin*-Cre mice show normal mechanical and heat nociceptive behavior

**Figure 5:**
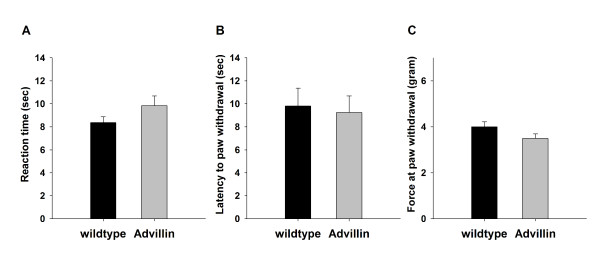
**Mechano- and heat nociceptive behaviour**. **A**. Reaction time in seconds of Advillin-Cre mice and their wildtype littermates during the hotplate test (p = 0.151). **B**. Latency to paw withdrawal in seconds during the dynamic plantar test (p = 0.699). **C**. Force applied at moment of paw withdrawal during the dynamic plantar test (p = 0.122). Data are expressed in mean ± SEM.

Litters (average n = 5-6) appeared healthy and had normal gross motor and sensory function, as assessed by the SHIRPA test [24]. By means of the hot plate test, we investigated acute thermal nociceptive thresholds (Figure 5A). The mean reaction time was comparable between Advillin-Cre mice (9.84 sec, n = 5) and their wildtype littermates (8.63 sec, n = 6). There was no significant difference between the two groups (p = 0.151) when compared using an unpaired *t*-test. In addition to thermal nociception, we assessed the sensitivity to mechanical nociceptive stimuli with an automated dynamic plantar test [25]. There was no significant difference in the mean paw withdrawal latency between Advillin-Cre mice and wildtype littermates (9.24 sec and 10.18 sec, respectively; p = 0.66) (Figure 5B). In addition, the mean force applied to induce a withdrawal response did not show significant differences between the two groups (Advillin-Cre: 3.5 g; wildtype littermates: 3.99 g; p = 0.122) (Figure 5C).

## Discussion

Based upon previous expression studies demonstrating that Advillin is expressed in a somatosensory-specific manner [8,9], we generated a BAC transgenic Cre-driver mouse line that expresses Cre-recombinase from the Advillin locus. We characterized this line by means of copy number analysis, X-gal staining and pain behavior. We found that Advillin is highly expressed in sensory ganglia such as TG and DRG, with sparser expression in vestibular ganglia, superior cervical ganglia, nodose ganglia, taste buds, secondary sensory nuclei in the brainstem, and the VTA in the midbrain. Finally, acute nociceptive responses to thermal and mechanical stimuli were detected to be normal.

Transgenic mice generated using BAC technology are widely used in biology to study gene function. BACs are excellent tools for maintaining large DNA fragments and are extensively used to generate transgenic Cre recombinase driver lines [26,27]. BAC-based transgenes have the advantage of usually directing gene expression at physiological levels with the same developmental timing and expression patterns as endogenous genes [28]. Generation thereof is less time consuming than embryonic stem (ES) cell mediated transgenesis by knock-in, with comparable end results [29]. With this in mind we chose to use a BAC transgenic-based approach to generate our Advillin-Cre transgenic mouse line. Of note, a Cre driver line has been described that utilized a knock-in strategy into the Advillin locus [30]. As yet, there is no reported characterization of the developmental or adult expression profile of Cre in these mice, nor any behavioral analysis to the best of our knowledge.

BAC transgenes usually contain sufficient regulatory information to recapitulate endogenous gene expression patterns [31], and the optimal transgene drives expression in a cell-specific, copy-dependent fashion [19,20]. Usually one to five BAC transgene concatemers integrate into a single locus of the genome [20,21,32] and an increased BAC transgene copy number correlates with increased BAC transgene expression [19,20]. However, rare cases have been reported, in which more than five copies were integrated [22,23]. Our copy number estimates are based on a standard curve of known BAC copy number standards, which is a useful means to confirm transgene expression and stable integration. Quantification by qPCR revealed that our Advillin-Cre mice carry five copies of the transgenes in their genomes. Based on a hallmark publication, copy numbers usually remain fixed in subsequent generations and among littermates [19].

We assessed the expression of the Cre gene in the Advillin locus using X-gal staining after breeding with R26R mice and a CFP reporter line. Functional Cre could be found in more than 82% of all neurons of these ganglia indicating that it was expressed by both, mechanoreceptors and nociceptors. The remaining Advillin-negative expressing neurons were not further characterized. However, secondary neurons of dorsal root afferents in the dorsal horn of the spinal cord were Advillin-negative, indicating that this Cre-line targets only the peripheral part of the somatosensory pathway. We were not able to detect Advillin-Cre expression in DRG at embryonic stages, while X-gal staining was clearly detectable in neonatal mice at P1. In previous studies, Advillin expression has been reported starting at embryonic stage E12.5 with peak expression between E14.5 and E16.5 [9,12]. Consistent with our negative spinal cord staining, expression of Advillin in the spinal cord has not been reported previously.

The trigeminal ganglion, which contains primary sensory neurons innervating the face, projects to trigeminal sensory nuclei (principal trigeminal nucleus and spinal trigeminal nucleus) in the brainstem (reviewed by [33]). We found X-gal staining also in these second-order neurons, however in adult mice only.

Although TG progenitors are generated at early developmental stages (E8.25 - E9) in the mouse, and brainstem nuclei progenitor cells are formed by stage E15.5 [33], we detected X-gal staining for functional Cre in trigeminal ganglia at embryonic stage E16.5 and E18.5 but not at E12.5. Advillin expression has been described at these earlier stages [9,12] and Hasegawa and colleagues [9] found scattered Advillin expression in TG at stage E11.5. In line with our study, they also reported adult Advillin expression in higher-order trigeminal nuclei [9].

In agreement with previous reports, we observed Advillin mediated Cre expression in the vestibular ganglion of adult mice [9]. In addition, we detected functional Cre expression in second-order brainstem neurons, and in the medial and spinal vestibular nuclei of adult mice. Moreover, during development, we observed Advillin expression in the vestibular ganglion at E18.5. Positive X-gal staining also occurred in taste buds of both the circumvallate and foliate papilla, which has been previously reported [12]. However, taste buds in the fungiform papillae, which are not innervated by the glossopharyngeal nerve but by the greater superficial petrosal nerve [34], were Cre negative. As for the TG and vestibular nuclei, we detected staining in second-order neurons of the gustatory pathway, in the nucleus of the solitary tract of the brainstem [34]. At embryonic stages, we did not detect any staining in these areas. The only other brain region where we found positive (albeit sparse) staining in adults was the ventral tegmental area (VTA) of the midbrain. This region contains mainly dopaminergic neurons and functions in the reward circuitry of the brain. From here, neurons project to numerous areas of the brain, such as the prefrontal cortex [35]. Besides the above-mentioned regions, other tissues and brain areas did not express functional Cre in our *Advillin*-Cre animals. In summary, Advillin is expressed in several sensory ganglia where it is found in primary and some secondary neurons. *Advillin*-Cre mice may therefore be useful driver lines for gene targeting in other sensory areas in addition to the somatosensory system.

To rule out a putative affect on nociceptive behavior response caused by expression of Cre in primary sensory neuron populations, we obtained data from paradigms testing thermal and mechanical nociceptive thresholds (hot plate test, dynamic plantar test) [36,37]. Responses to these stimuli were similar in Advillin-Cre mice and their wildtype littermates (Figure 5A). In addition, transgenic animals had normal overall gross motor and sensory function, assessed by the SHIRPA test [24]. Thus our data indicate that expression of Cre from the Advillin locus does not alter baseline behavioral responses.

Taken together, this is the first report of a sensory neuron specific Cre driver line with expression covering both mechanoreceptive and nociceptive neurons. Furthermore the late developmental onset of Cre expression will be advantageous in avoiding developmental defects and lethality associated with some genes. Advillin-Cre mice have been deposited at the EMMA mouse repository.

## Conclusions

Expression of Cre recombinase from the Advillin locus is apparent in almost all peripheral sensory neurons of the DRG and TG. In addition, Cre-recombination is also evident in some vestibular and gustatory neurons and in second order neurons of the brainstem. Thus, Advillin Cre mice are an important addition to the genetic toolbox for conditional gene ablation studies in sensory biology.

## Materials and methods

### Generation of transgenic construct and ET recombination

A 175-kb bacterial artificial chromosome (BAC) containing the mouse Advillin locus (RP23-109K12) was obtained from a C57BL/6J BAC library (Children's Hospital Oakland Research Institute). Individual BAC clones were thoroughly characterized and subsequently transformed with a plasmid conferring competence for lambda RedET mediated recombineering. A cassette containing the SV40-large T antigen nuclear localization signal, the Cre cDNA, a V5 epitope tag (Invitrogen), a SV40 T polyadenylation site, and a selectable marker (a kanamycin cassette under the prokaryotic em7 promoter) was assembled in a pBSSK^+ ^backbone. Insertion of the NLS-Cre-V5-pA cassette into the BAC backbone was performed using RedET recombineering as previously described [15,16], and targeted clones were selected for growth on Kanamycin. Clonal isolates were re-streaked twice and clonal identity was confirmed by PCR, restriction-digestion and full length sequencing of the inserted cassette. Purified BAC DNA was dissolved into Endotoxin-free TE and injected into pronuclei derived from the hybrid B6D2F1/N strain (Charles River Laboratories) or prepared for intracytoplasmic sperm injection (ICSI) as previously described [17,18]. Both methods were successful and produced offspring. Mice progeny were analyzed for BAC insertion by genomic PCR amplification with the following primer: *5'gcactgatttcgaccaggtt3' *and *5'gagtcatccttagcgccgta3'*.

### Copy number analysis

Real-time PCR (LightCycler^® ^480 SYBR Green I Master kit, Roche Applied Science) with Cre genotype primers was used to estimate the BAC transgene copy number in BAC transgenic lines. Advillin-Cre BAC cDNA was spiked into WT genomic DNA at 1, 2, 4, 8, 16, and 32 copies per genome. A standard curve was calculated from the crossing points of spiked DNA and compared to results of Advillin-Cre mouse genomic DNA.

### X-gal staining for β-galactosidase (*lacZ*) activity

#### Animals and tissue preparation

Heterozygous male Advillin-Cre^tg/+ ^mice were mated with the homozygous female Rosa26-*lacZ^tg/tg ^*(R26R) reporter mouse strain [14] (Jackson Laboratories, Bar Harbor, ME). Embryos were collected at E12.5, E16.5, and E18.5 for developmental analysis. Additionally, neonatal (postnatal day 1, P1) mice and tissues from adult mice were investigated. Mouse embryos and adult tissues were dissected in the presence of PBS containing 0.9 mM CaCl_2 _and 0.5 mM MgCl_2_. After genotyping for a double expression of Advillin-Cre and *lacZ*, embryos and adult tissues were stained for β-galactosidase expression (whole-mount or cryostat sections of 20 μm).

#### X-gal staining

Tissues and sections were stained with X-gal for *lacZ *activity as described in [38,39]. Briefly, after dissection embryos, tissues and sections were rinsed in phosphate buffer (0.1 M sodium phosphate monobasic, 0.1 M sodium phosphate dibasic, pH 7.3) for 10 minutes. They were fixed in 4% formaldehyde dissolved in ddH_2_0 for 10-30 minutes at room temperature and rinsed three times for 30 minutes in wash buffer (2 mM MgCl_2_, 0.01% deoxycholate, 0.02% Nonidet-P40, and 100 mM NaPO_4_, pH 7.3). Tissue and sections were stained for 2 hours or overnight at 37°C in wash buffer containing 1 mg/ml X-gal, 6 mM potassium ferrocyanide, and 5 mM potassium ferricyanide.

### Quantification of Advillin-Cre expression

We crossed male Advillin-Cre^tg/+ ^mice with a CFP reporter mouse strain. DRG were frozen in tissue embedding compound (OCT) directly after isolation. 12 μm thick sections were cut with a cryostat and dried at room temperature for 10 min. Afterwards, sections were postfixed in cold 4% formaldehyde on ice for 5 min and washed 10 min in PBS on ice. Standard immunefluorescence protocols were applied for the staining with chicken anti-GFP (1:500 concentration, Aves Labs, GFP-1020) and mouse anti-NeuN (1:100 concentration, Millipore MAB377). Sections were background corrected by comparison to control sections only labeled with secondary antibodies and counted for NeuN and GFP staining.

#### Imaging of stained tissue and sections

Whole-mount tissue images were taken with a stereomicroscope (MacroFluo Leica Z16A, CCD camera CD500). Mosaic images of sections were taken with a motorized widefield microscope (Leica DMRXA, high resolution colored CCD camera DC300F).

### Behavioral assessment of nociception

#### Animals

Five to six heterozygous male transgenic Advillin-Cre^tg/+ ^animals and their wild-type littermates were used at age two to three months. Animals were housed in groups of 3-4 per cage with food and water *ad libitum *at 22-24°C on 12-hour light-dark cycle. All mice were acclimated for at least 1 week prior to testing. All tests were carried out between 13:30 and 15:30 where mice were habituated 30 minutes prior to the testing room. All procedures were approved by the Italian Ministry of Health and the EMBL Animal Ethics Committee.

#### Modified SHIRPA Test

The modified SHIRPA is a primary screening based on observational assessment [24]. It is a basic behavioral battery that includes measures of muscle, cerebellar, sensory, and autonomic functions. It allows detecting overt, serious dysfunctions in the mouse before further behavioral phenotyping takes place.

#### Thermal Nociception

Pain sensitivity to thermal stimulus was tested using the hot plate test [37]. We recorded the hind-paw withdrawal latency to heat stimulation using a Hot Plate apparatus (Ugo Basile, Varese, Italy). Briefly, mice were individually placed on a hot plate with the temperature adjusted to 55 ± 1°C. The time to the first sign of nociception was recorded and the animal was immediately removed from the hot plate. A cut-off period of 60 seconds was maintained to avoid damage to the tissue. Hind-paw response was scored from videotape as either a foot lifting (jump) or a foot shake or a paw lick [36,40]. Each mouse was used once only.

#### Mechanical Nociception

Mechanical allodynia was assessed using a Dynamic Plantar Anesthesiometer (Ugo Basile, Italy) by measuring the latency to withdraw the hind paw from a graded force applied to the plantar surface using a von Frey filament.

The electronic *von Frey *device applied a single non-flexible filament (0.5 mm in diameter) with increasing force (0.1 g/s; from 0 to 5 g, [25]) against the plantar surface over a 10-second- period. The paw withdrawal response automatically turned off the stimulus, and the pressure eliciting the response was recorded. For measurements, mice were placed individually into red enclosures on a framed metal mesh floor and allowed to acclimate for 10 minutes before testing. Paw withdrawal thresholds were measured in triplicate for each paw of each animal, allowing at least 30 second intervals between successive measurements. In case of no withdrawal up to 5 grams this maximum force is maintained until the paw is withdrawn. The paw withdrawal threshold is numerically obtained in grams (force applied) and in seconds (latency of withdrawal).

#### Data and statistics

All data are presented as mean ± SEM. Unpaired *t*-tests were applied and p < 0.05 was considered statistically significant.

## Competing interests

The authors declare that they have no competing interests.

## Authors' contributions

All authors read and approved the final manuscript. PAH designed experiments. AF and EP performed immunohistochemistry and SZ assisted with experiments. SZ supervised the project and wrote the manuscript with discussion from all authors. PH created the construct. MAB performed behavioral analyses of animals. PNM performed the pronuclear injection and ICSI. CM did the copy number analysis.
